# Phylogenomics of five *Pseudanabaena* cyanophages and evolutionary traces of horizontal gene transfer

**DOI:** 10.1186/s40793-023-00461-5

**Published:** 2023-01-13

**Authors:** Jie Zhu, Feng Yang, Kang Du, Zi-Lu Wei, Qing-Fa Wu, Yuxing Chen, Wei-Fang Li, Qiong Li, Cong-Zhao Zhou

**Affiliations:** School of Life Sciences, University of Science and Technology of China, Hefei, 230027 Anhui China

**Keywords:** Freshwater cyanophage, Cyanobacterium, *α*-proteobacterium, Horizontal gene transfer, Evolutionary trace, Lake Chaohu

## Abstract

**Background:**

Along with the fast development and urbanization in developing countries, the waterbodies aside the growing cities become heavily polluted and highly eutrophic, thus leading to the seasonal outbreak of cyanobacterial bloom. Systematic isolation and characterization of freshwater cyanophages might provide a biological solution to control the awful blooms. However, genomic sequences and related investigations on the freshwater cyanophages remain very limited to date.

**Results:**

Following our recently reported five cyanophages Pam1~Pam5 from Lake Chaohu in China, here we isolated another five cyanophages, termed Pan1~Pan5, which infect the cyanobacterium *Pseudanabaena* sp. Chao 1811. Whole-genome sequencing showed that they all contain a double-stranded DNA genome of 37.2 to 72.0 kb in length, with less than half of the putative open reading frames annotated with known functions. Remarkably, the siphophage Pan1 encodes an auxiliary metabolic gene *phoH* and constitutes, together with the host, a complete queuosine modification pathway. Proteomic analyses revealed that although Pan1~Pan5 are distinct from each other in evolution, Pan1 and Pan3 are somewhat similar to our previously identified cyanophages Pam3 and Pam1 at the genomic level, respectively. Moreover, phylogenetic analyses suggested that Pan1 resembles the *α*-proteobacterial phage vB_DshS-R5C, revealing direct evidence for phage-mediated horizontal gene transfer between cyanobacteria and *α*-proteobacteria.

**Conclusion:**

In addition to the previous reports of Pam1~Pam5, the present findings on Pan1~Pan5 largely enrich the library of reference freshwater cyanophages. The abundant genomic information provides a pool to identify novel genes and proteins of unknown function. Moreover, we found for the first time the evolutionary traces in the cyanophage that horizontal gene transfer might occur at the level of not only inter-species, but even inter-phylum. It indicates that the bacteriophage or cyanophage could be developed as a powerful tool for gene manipulation among various species or phyla.

**Supplementary Information:**

The online version contains supplementary material available at 10.1186/s40793-023-00461-5.

## Background

Bacteria could acquire foreign genetic materials from other organisms or the surrounding environments, via horizontal gene transfer (HGT), to obtain a variety of novel traits, including antibiotic resistance, virulence factors and so on [1]. The frequent HGT events enable bacteria to explore or adapt to new habitats, and hence facilitates the microbial evolution [2–4]. As the most abundant entities on the Earth, the bacteriophages serve as a group of efficient vectors of HGT, capable of packaging and transferring foreign or their own DNA from the host to another bacterium, the process of which is termed transduction [5]. In fact, the viral DNA sequences have been found in ~30% of publicly available bacterial genomes [6]. Compared to the other two classic mechanisms of HGT—transformation and conjugation, which can transfer DNA across large phylogenetic distance, transduction is usually constrained in a rather narrow host range of bacteriophages and further restricted by the ecological barrier [7, 8].

Cyanophages, which are widespread in various waterbodies, are a group of bacteriophages that specifically infect and lyse cyanobacteria [9]. Cyanobacteria belong to a phylum of ancient photosynthetic microbes that have lived on Earth for ~3.5 billion years [10]; however, their uncontrollable growth usually causes severe and harmful algal blooms in freshwater and marine ecosystems [11]. The co-evolution with the host and global regulation on the population and community of cyanobacteria make the cyanophage a potential eco-friendly and bio-safe agent to prevent and control the cyanobacterial blooms [12]. Except for the two so-called tail-less cyanophages PaV-LD [13] and PA-SR01 [14], the cyanophages are classified in the tailed dsDNA bacteriophage *Caudovirales*, which are generally grouped into three families according to the tail morphology: *Myoviridae* with a long contractile tail, *Podoviridae* with a short noncontratile tail, and *Siphoviridae* with a long flexible tail [15]. To date, approximately 180 cyanophages have been genome-sequenced, most of which were isolated from the marine cyanobacteria, leaving very limited information on freshwater cyanophages.

The cyanophages play a crucial role in not only the global biogeochemical cycle of carbon, oxygen, and nitrogen [16], but also co-evolution with the cyanobacterial hosts [17], via phage-host interactions and exchange of genes. A study on T7-like cyanophages infecting marine *Synechococcus* and *Prochlorococcus*, the dominant primary producers in the oceans, showed that cyanophages from the less aggressive and more diverse clade have greater ecological impacts compared to their sister clade [18]. During infection, cyanophages could acquire auxiliary metabolic genes (AMGs) from the hosts, which would augment key steps in host metabolism, and/or increase phage replication and progeny release [19]. A variety of AMGs involved in photosynthetic pathways have been identified from the genomes of cyanophages in various environments, such as genes of photosystem, phycobilin biosynthesis and phycobilisome degradation [20–22]. Compared to the marine counterparts, freshwater cyanophages possess relatively fewer detectable homologues of host-derived AMGs, which might suggest an alternative mechanism to maintain host metabolism and in turn the efficient viral reproduction [23]. Moreover, the limited information of freshwater cyanophages restricts comprehensive understanding on their ecology and evolution.

Lake Chaohu, one of the five largest freshwater lakes in China, suffers from serious cyanobacterial blooms every summer, due to industrial and domestic pollution from the fast-developing city Hefei and the constantly released phosphorus from the neighboring phosphorite mine. Recently, we have identified and sequenced five freshwater cyanophages Pam1~Pam5 from Lake Chaohu, which enabled us to assemble ten uncultured cyanophages from the corresponding metagenomic data [24]. Using a strain of cyanobacterium *Pseudanabaena* sp. Chao 1811 from Lake Chaohu as the host, here we further isolated and purified four long-tailed and one short-tailed freshwater cyanophages, which are termed Pan1~Pan5. Comparative genomic and phylogenetic analyses suggested that these five cyanophages are evolutionarily distinct from each other, despite sharing a same cyanobacterial host. In addition to a series of evolutionary traces of HGT among Pan1~Pan5 and our previously reported Pam1~Pam5, we revealed that Pan1 might function as a vector of phage-mediated inter-phylum HGT between its host *Pseudanabaena* and the α-proteobacteria that usually co-existed in a same community.

## Methods

### **Isolation and identification of*Pseudanabaena*sp. Chao 1811**

The *Pseudanabaena* sp. Chao 1811 was isolated from the water sample of the Nanfei estuary towards Lake Chaohu collected in November, 2018. The colony was iteratively selected from the BG-11 solid plate three times, and cultured in the BG11 medium. The cells were grown at 25 °C to an OD_680 nm_ of 0.6~0.8 under a light intensity of 6~27 µmol photons m^− 2^s^− 1^ in a 14-hr light/10-hr dark cycle. Optical microscopy was used to observe the morphology of *Pseudanabaena* sp. Chao 1811.

Moreover, the genomic DNA was extracted and applied to the 16S rRNA and whole-genome sequencing, respectively. Whole-genome sequencing was performed using the Next-Generation Sequencing strategy under the Illumina HiSeq platform combined with the Single Molecule Real-Time strategy under the PacBio Sequel II platform (Tianjin Biochip Corporation).

The reads of 16S rRNA sequencing were further used to calculate the microbial diversity at the class level, to reveal the co-existed bacteria with *Pseudanabaena* sp. Chao 1811. One *α*-proteobacterial contig was assembled from the whole-genome raw sequencing data of *Pseudanabaena* sp. Chao 1811 via SPAdes software [25].

### Screening and purification of cyanophages Pan1~Pan5

Using double-layer plaque assays, five cyanophages were isolated from the surface water samples collected in June 2018, at the Nanfei estuary of Lake Chaohu, which could infect a same host *Pseudanabaena* sp. Chao 1811. The crude lysate was treated with 1 µg/mL DNase I and RNase at room temperature for 1 h, and NaCl was added to the solution at a final concentration of 0.5 M, followed by incubation on ice for 2 h. After 10 min of centrifugation at 8,000 g, the supernatant was further treated with 10% polyethylene glycol 6,000 at 4 °C for 15 h. The cyanophage particles were pooled by centrifugation (8,000 g, 4℃, 20 min), and then resuspended in SM buffer (50 mM Tris, pH 7.5, 10 mM MgSO_4_, 100 mM NaCl). Subsequently, the phage particles were further purified by cesium chloride density gradient centrifugation at 100,000 g for 4 h (SW41 Ti rotor, Beckman Coulter, Indianapolis, IN, USA), and dialyzed against SM buffer. In sum, five strains of cyanophages were isolated and purified, which were sequentially termed Pan1~Pan5 after their host.

### Morphologies of cyanophages Pan1~Pan5

The morphologies of cyanophages Pan1~Pan5 were characterized by negative-stain transmission electron microscopy. First, a drop of 3 µL samples containing the purified cyanophages was loaded onto a hydrophilized carbon-coated copper grid, which was then incubated with 2% uranyl acetate for 1 min. The negatively stained viral particles were observed with an FEI Tecnai G2 120 kV transmission electron microscope.

### Extraction of phage genomic DNA and the whole-genome sequencing

The cyanophage particles were first treated with DNase I and RNase at a final concentration of 10% at 37 °C for 1 h. An equal volume of 2× lysis buffer (20 mM EDTA and 0.5% SDS) was added to the solution, which was further incubated with 20 mg/mL proteinase K at 56 °C for 2 h. The phage suspension was sequentially treated with equal volume of balanced phenol, balanced phenol–chloroform (1:1) and chloroform, respectively. Then, 0.3 M sodium acetate was added to the solution, followed by a 3-fold volume of ethanol at −80 °C for 5 h. The precipitated phage genomic DNA was washed twice with 70% ethanol, and finally resuspended in sterile water that has been heated to 56 °C. The extracted genomic DNA was sequenced based on an Illumina MiSeq or Novaseq platform (Shanghai Personal Biotechnology Co., Ltd, China), after constructing the library according to the Illumina TruSeq DNA Sample Preparation Guide. After removing the adapters and poor-quality reads, the softwares SPAdes [25] and A5-MiSeq [26] were used to *de novo* assemble the genomes, and Pilon [27] was applied to correct the assembled contigs.

### Genome annotation and comparison

GeneMarkS [28] was used to predict the putative open reading frames (ORFs) of each cyanophage, whose function was predicted by BLASTp (https://blast.ncbi.nlm.nih.gov) against the nr protein database in NCBI. The results with an e-value < 10^− 3^ are considered believable, of which hits with the minimal e-value are regarded as orthologs. Alternatively, HHpred [29] was used to annotate the putative ORFs. In addition, AlphaFold2 [30, 31], in combination with the Dali sever [32, 33], was also applied to predict the structural proteins. The circular genome maps were drawn with Proksee (https://proksee.ca).

### Construction of proteomic trees

ViPTree (http://www.genome.jp/viptree) was used to construct viral proteomic trees and perform whole-genome alignments. The genome-wide sequence similarity distance was calculated via tBLASTx, and bionj tree based on the distance (1-similarity) matrix was then constructed [34]. The calculation of genomic similarity score (SG) and details of the methodology could be found in previous reports [34, 35]. The cutoff of the SG for the clustering was set to 0.15 at a viral genus-level. The SG value of 1 stands for two identical genomes, whereas that of 0 represents no detectable high-scoring segment pairs by tBLASTx. The complete genomes of 183 previously reported cyanophages were used in Fig. 3 to investigate the evolutionary relationship of Pan1~Pan5 in cyanophages, whereas the genomes of 4918 dsDNA bacteriophages were used in Fig. 4A to explain the evolutionary origins of Pan1~Pan5 at a much global view.

### Alignments of homologous proteins and the related phylogenetic analyses

The encoded proteins of cyanophage Pan1 and bacteriophage vB_DshS-R5C were respectively aligned with those of their hosts *Pseudanabaena* sp. Chao 1811 and *Dinoroseobacter shibae* DFL 12 via the BLASTp program [36]. The proteins between phage and host with an e-value of < 0.05 are regarded as homologs. Multiple sequence alignments of the phosphate starvation-induced protein (PhoH) from cyanobacteria and *α*-proteobacteria were performed using ClustalW. Then, the phylogenetic tree was constructed with MEGA 11 [37], using the default parameters with a bootstrap value of 1000, and the maximum likelihood method. In total, 56 amino acid substitution models were applied to find the correct evolutionary model for PhoH: the “LG + G + I” model, which possesses the lowest Bayesian Information Criterion (BIC) score. The phylogenetic tree was finally displayed by iToL [38].

### **Assembly of an α-proteobacterial contig from the whole-genome sequencing data of*Pseudanabaena*sp. Chao 1811**

The clean reads from the whole-genome sequencing data of *Pseudanabaena* sp. Chao 1811 under the PacBio Sequel II platform were assembled by Canu v2.2 software [39] with the genome size setting as 5 M, followed by three-round correction via Pilon v1.24 software [27] based on the raw sequencing data under the Illumina HiSeq platform. Using BLASTn v1.12.0 [36], the contigs belonging to the genome and plasmids of *Pseudanabaena* sp. Chao 1811 were identified, which were further aligned with the raw whole-genome sequencing data by Minimap2 v2.22 [40]. The unaligned reads in the raw sequencing data were extracted using samtools v1.13 [41], and applied to a second-round assembly, which was also performed by Canu v2.2 [39] and Pilon v1.24 [27]. According to the results of BLASTn v1.12.0 [36] analysis, one of the assembled contigs with a length of 3,135,941 bp was identified as the circular genome of *α*-proteobacteria—*Sphingobium*, which was further annotated via the program Prokka v1.14.6 [42]. In addition, via BLASTp v1.12.0 [36], the translated ORFs of the α-proteobacterial contig were searched against those of *Pseudanabaena* sp. Chao 1811 with an e-value of < 10^− 3^.

## Results

### **Identification of*Pseudanabaena*sp. Chao 1811 and five cyanophages**

**Fig. 1:**
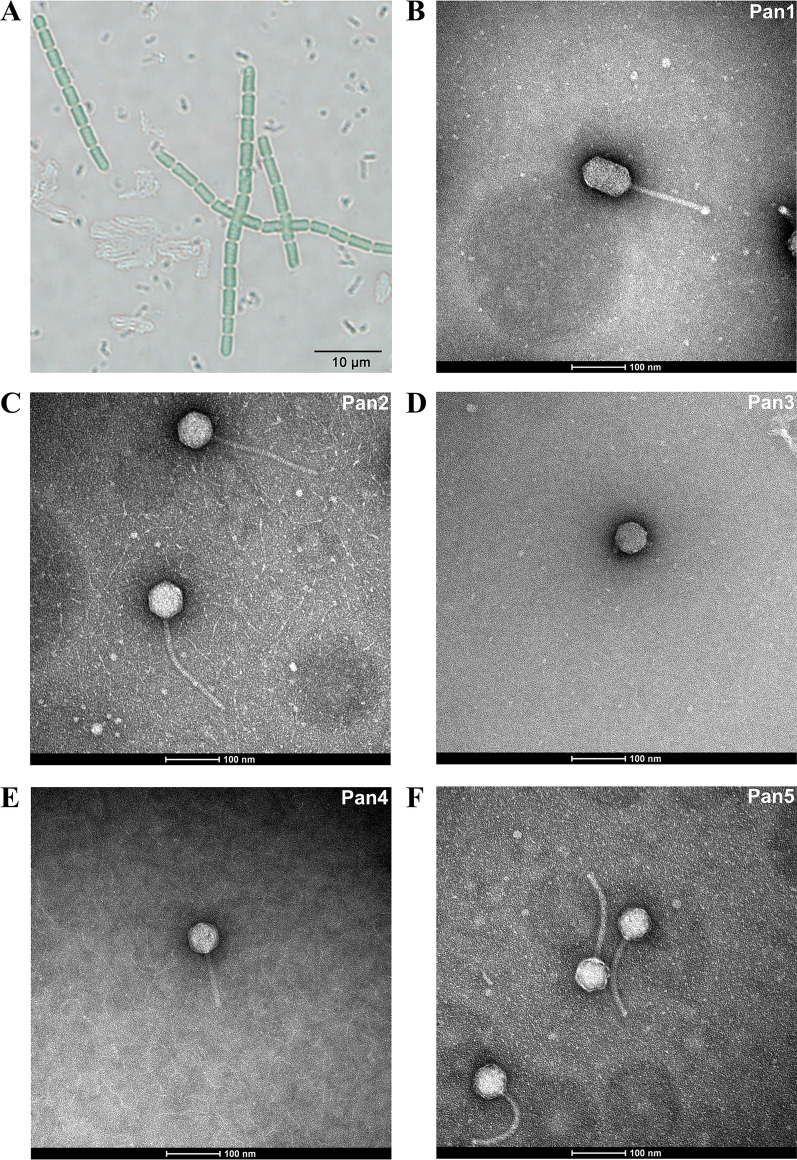
Identification of *Pseudanabaena* sp. Chao 1811 and five cyanophages. **A** The image of *Pseudanabaena* sp. Chao 1811 photographed by inverted optical microscopy. It possesses a typical morphology similar to those previously reported *Pseudanabaena* strains [14]: (1) cell division in one plane and intercellular breakage of trichome (filament); (2) straight trichomes composed of multiple barrel-shaped cells; (3) cells with a length bigger than the diameter; (4) cell walls constricted at the cell junction. The scale bar is 10 μm. **B–F** Negative-staining images of the purified Pan1~Pan5 particles under a 120 kV transmission electron microscope. Pan1 has a prolate icosahedral head of 94 nm in length and 58 nm in width, whereas Pan2~Pan5 all possess a classic icosahedral head of about 60~70 nm in diameter. Except for the short-tailed Pan3, Pan1, 2, 4 and 5 have a long and flexible tail in length of 144, 210, 100 and 149 nm, respectively

After three rounds of screening, a strain of cyanobacterium was isolated from the water sample at the Nanfei estuary towards Lake Chaohu collected in November, 2018. Based on the 16S rRNA sequences, this cyanobacterial strain was assigned to the genus *Pseudanabaena*, and termed *Pseudanabaena* sp. Chao 1811. Indeed, it adopts a typical morphology of *Pseudanabaena* as observed by optical microscopy (Fig. 1A). The whole-genome sequencing showed that *Pseudanabaena* sp. Chao 1811 possesses a genome of 4,729,581 bp with a G + C content of 43%, which harbors 4,329 putative open reading frames (ORFs).

Using *Pseudanabaena* sp. Chao 1811 as the host, five strains of cyanophages were isolated and purified from the surface water samples of Lake Chaohu, which were sequentially termed Pan1~Pan5. The negative-stain transmission electron microscopy showed that Pan1 possesses a prolate icosahedral head of 94 nm in length and 58 nm in width, which resembles bacteriophage T4 [43], whereas Pan2~Pan5 all have a classic icosahedral head of about 60~70 nm in diameter (Fig. 1B–F,  Additional file 1: Table S1). Based on the tail morphology, they belong to two families: the siphophages Pan1, 2, 4, 5 with a long and flexible tail of 144, 210, 100 and 149 nm, respectively, and the podophage Pan3 with a short and non-contractile tail (Fig. 1B–F,  Additional file 1: Table S1).

### Genomic sequences of cyanophages Pan1~Pan5

**Fig. 2:**
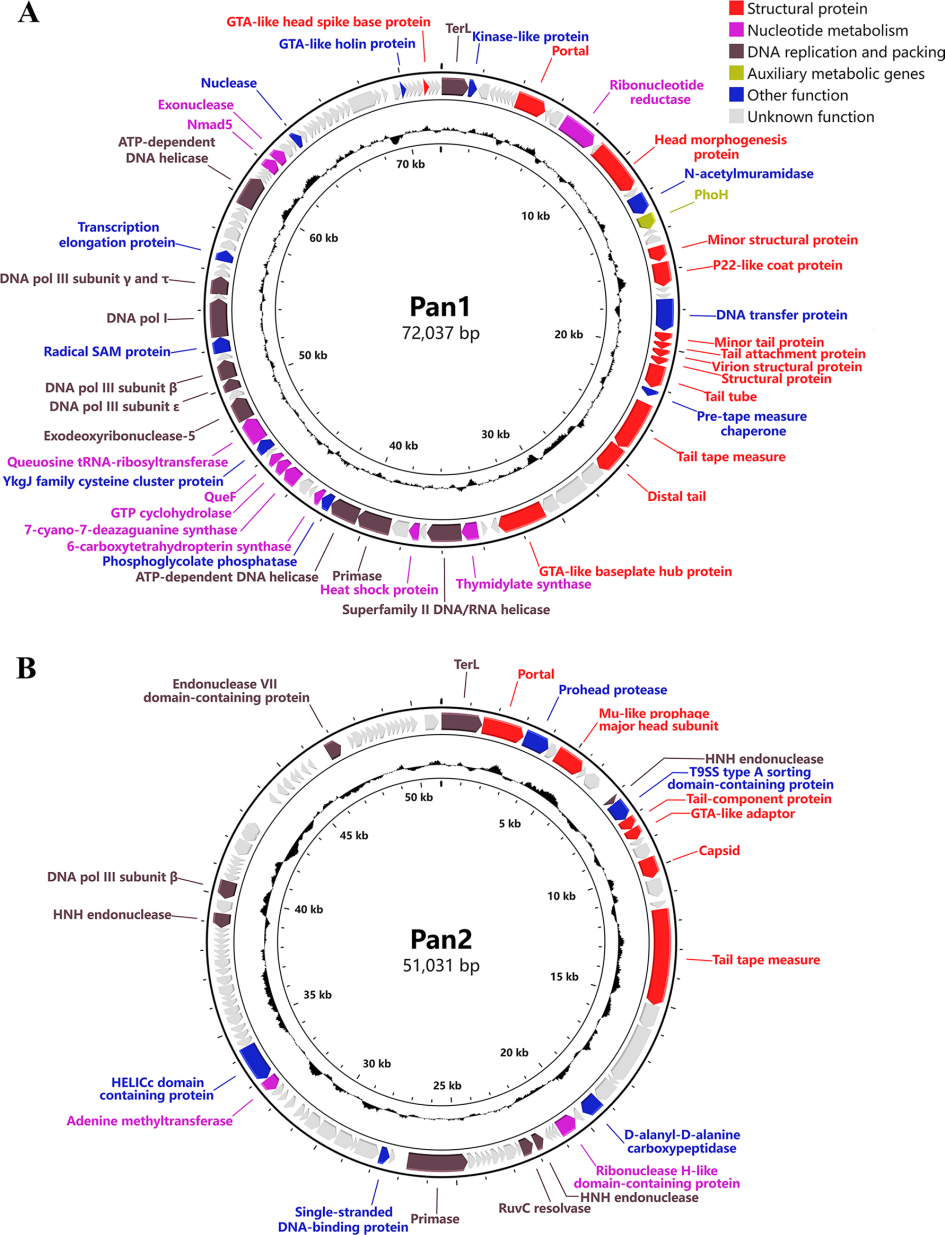

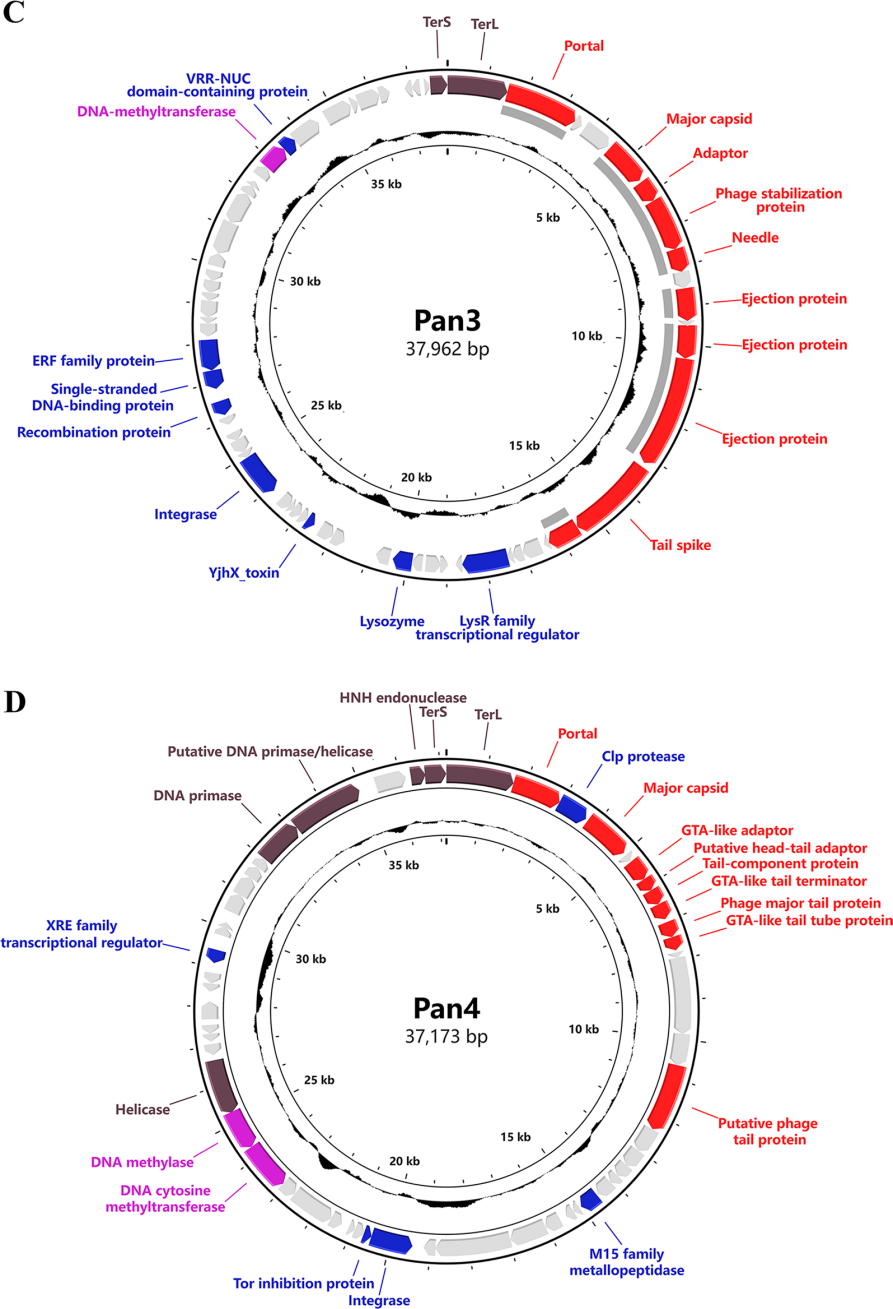

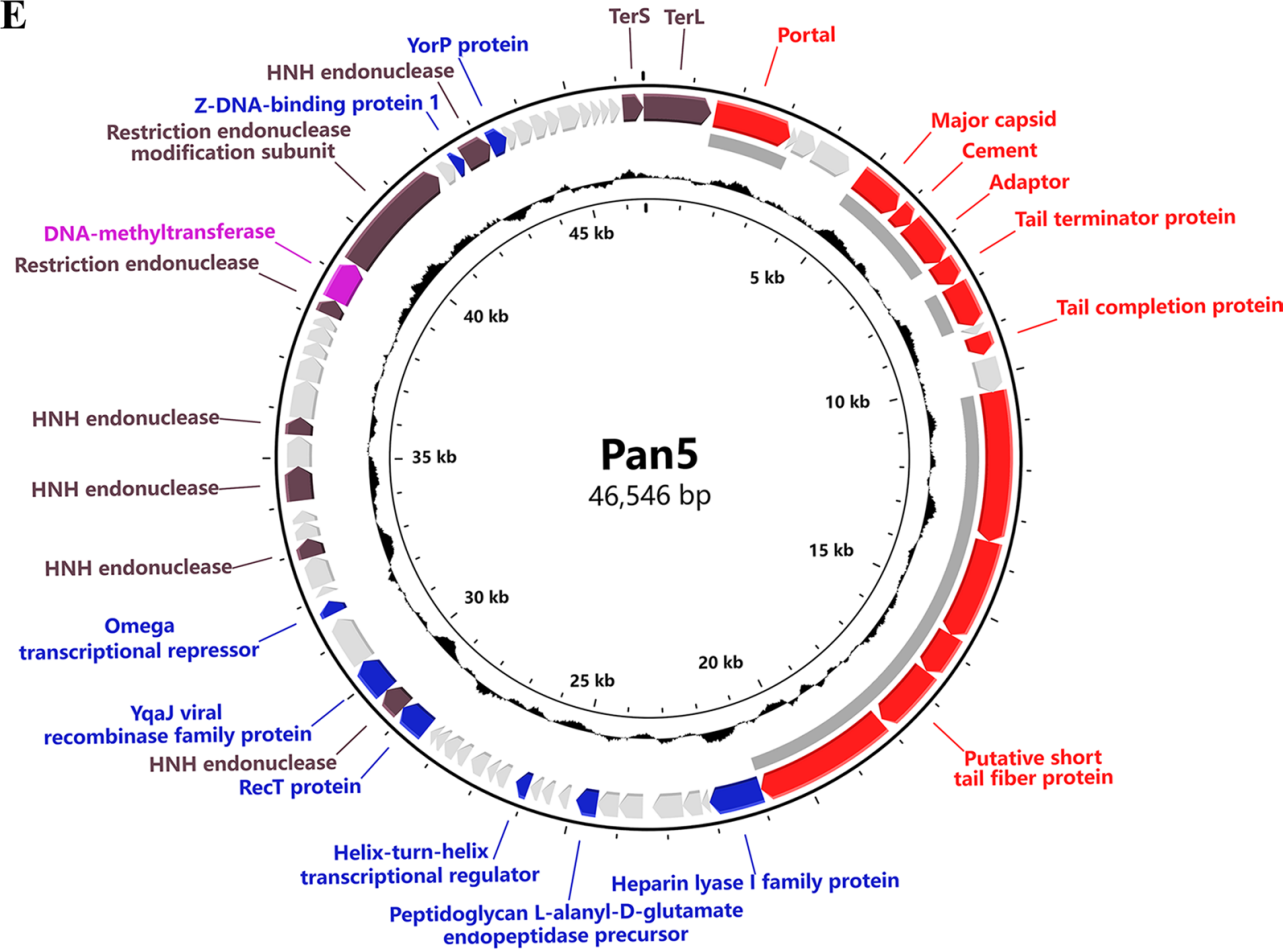
The circular genomic maps of **A–E** cyanophages Pan1~Pan5, respectively. Circles from the outermost to the innermost represent the following: (i) predicted ORFs with known functions are labeled and colored based on their functions; (ii) structural proteins identified by mass spectrometry shown by gray lines; and (iii) GC content plotted relative to the genomic mean of 35% G + C

The genomic DNA of cyanophages Pan1~Pan5 were respectively extracted and applied to whole-genome sequencing. The assembly results showed that these five cyanophages Pan1~Pan5 all contain a double-stranded DNA genome, in length of 72,037, 51,031, 37,962, 37,173 and 46,546 bp, respectively (Fig. 2). Except for that of Pan5, the G + C contents of Pan1~Pan4 are significantly higher than that of the host *Pseudanabaena* sp. Chao 1811 (Additional file 1: Table S1). Notably, Pan1, which has a prolate head, possesses a highly compact genome with a genome size per volume of 0.72 bp/nm^3^, suggesting a unique genome packaging pattern (Additional file 1: Table S1).

Moreover, GeneMarkS analyses [28] showed that cyanophages Pan1~Pan5 possess 104, 103, 65, 55 and 71 ORFs, respectively (Additional file 1: Table S2). Further genome annotation by BLASTp (https://blast.ncbi.nlm.nih.gov) and HHpred [29] indicated that less than half of the putative ORFs have known functions (Additional file 1: Table S2), leaving most ORFs hypothetical. The annotated proteins of Pan1~Pan5 could be divided into four functional groups: structural proteins, nucleotide metabolism, DNA replication and packing, and other function (Fig. 2, Additional file 1: Table S2); besides, a canonical AMG *phoH* (*gp18*) was assigned in Pan1 (Fig. 2 A). In addition, we found for the first time in cyanophages that Pan1 encodes a DNA transfer protein gp25, which contributes to the injection of phage DNA into the host [44].

In the genome of Pan1, five genes encoding gp48, gp51-53 and gp56 were annotated to the conserved pathway of tRNA queuosine modification in bacteria (Additional file 1: Fig. S1, Table S3). In fact, an almost complete queuosine modification pathway could be identified in the genome of host *Pseudanabaena* sp. Chao 1811, including ORF0224/QueC, ORF2946/QueF, ORF2528/TGT, and ORF3562/GCH, which are homologous to gp51-53 and gp56, respectively, in addition to ORF0223/QueE, ORF0762/QueG and ORF2435/QueA (Additional file 1: Fig. S1, Table S3). Notably, the homologous protein of Pan1 gp48/QueD, which is responsible for the synthesis of the queuosine-tRNA biosynthetic intermediate 7-cyano-7-deazaguanine [45–47], is absent in the host genome. It suggested that Pan1 that encodes an extra salvage synthase QueD, together with its host, constitutes a complete pathway for queuosine modification.

Further search against the genomes of Pan1~Pan5 via DRAM-v [48] showed that except for *phoH* (*gp18*) of Pan1, which has been well characterized within marine cyanophages, additional five genes of Pan1 and one gene of Pan4 are predicted as AMGs. Among them, *gp48* and *gp51*-*gp53* of Pan1 are involved in the aforementioned queuosine tRNA modification (Additional file 1: Fig. S1), whereas *gp13* of Pan1 and *gp35* of Pan4 participate in DNA synthesis and modification. All these AMGs might help Pan1 and Pan4 modulate host cell metabolism during infection for the efficient phage replication and reproduction.

### Comparative genomic and proteomic analyses of Pan1~Pan5

**Fig. 3:**
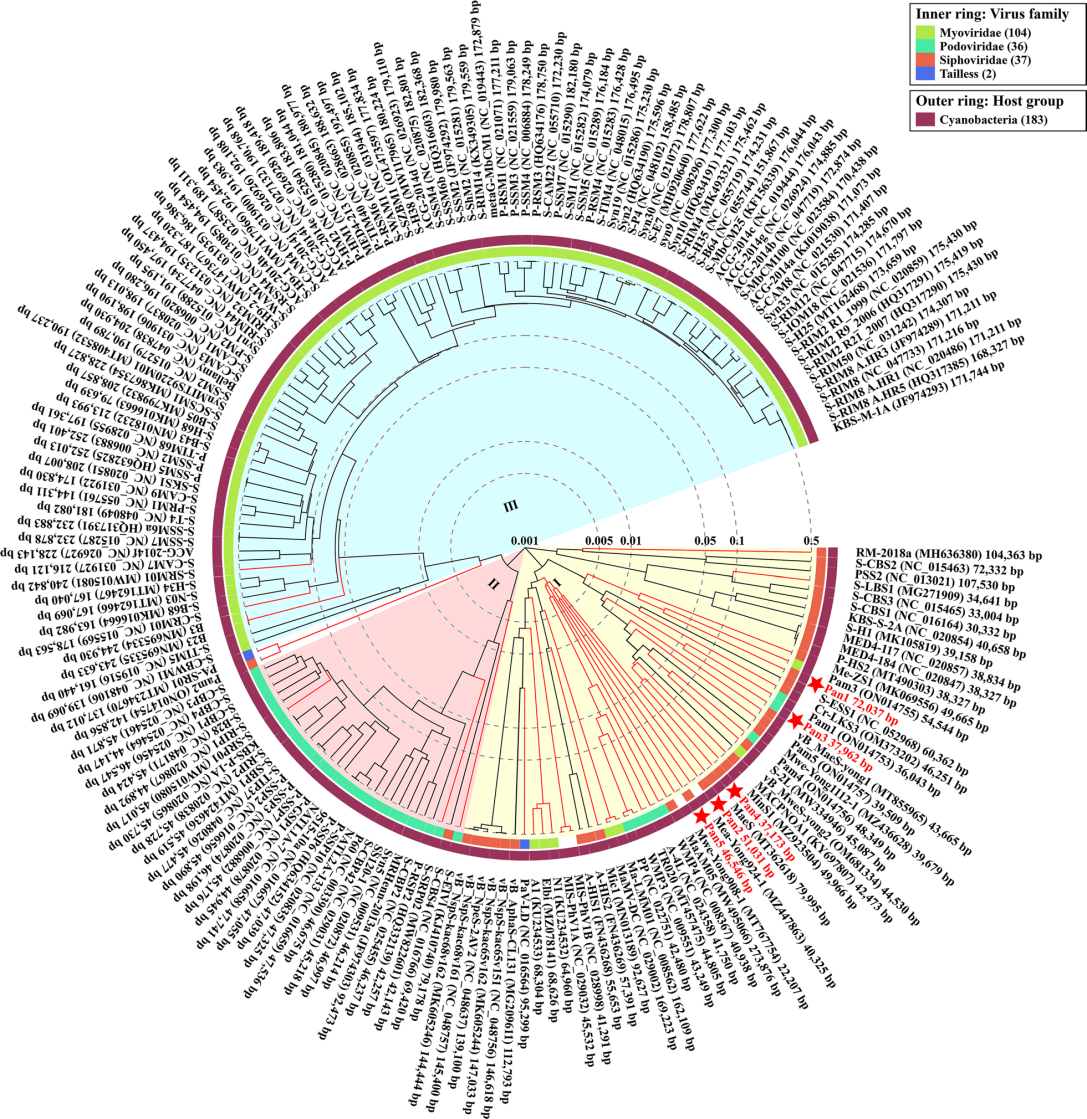
The proteomic tree of cyanophages Pan1~Pan5. The tree was constructed based on the complete genomes of 183 previously reported cyanophages by ViPTree [34]. In addition to 120 genome sequences downloaded from the Virus-Host Database (https://www.genome.jp/virushostdb/), the remaining 63 sequences were obtained from the GenBank database (https://www.ncbi.nlm.nih.gov/genbank/) and manually added to the ViPTree to construct the proteomic tree. The freshwater cyanophages are colored in red, whereas Pan1~Pan5 are marked with a red star, respectively. Cyanophages in the three clusters are labeled in different colors: (I) marine *Siphoviridae* and freshwater cyanophages (yellow), (II) marine *Podoviridae* (pink), and (III) marine *Myoviridae* (blue)

Comparative analyses showed that Pan1~Pan5 differ from each other at the genomic level, except that the genome of Pan1 shares a dozen of homologous segments at a sequence identity of < 50% with those of Pan4 (Additional file 1: Fig. S2). Moreover, to further investigate the evolutionary relationship of these five cyanophages, a proteomic tree was performed via ViPTree [34] against the genomes of 183 sequenced cyanophages, including the six strains of cyanophages that infect the genus *Pseudanabaena*—PA-SR01 isolated from a reservoir of Singapore [14] and our previously isolated cyanophages Pam1~Pam5 from Lake Chaohu [24]. Based on the normalized SG [49], the cyanophages were grouped into three clusters: cluster I containing marine long-tailed cyanophages and the majority of freshwater cyanophages, in addition to the short-tailed and contractile-tailed cyanophages from marine in clusters II and III, respectively (Fig. 3).

The proteomic tree of 183 cyanophages showed that Pan1~Pan5 were located at two individual branches of 0.001 < SG < 0.005: one with Pan1, Pan3 and Pan4, and the other with Pan2 and Pan5 (Fig. 3). However, *Siphoviridae* Pan1 and *Podoviridae* Pan3 share a close evolutionary distance with our previously isolated *Pseudanabaena* cyanophages *Myoviridae* Pam3 and *Podoviridae* Pam1, of 0.01 < SG < 0.05 and 0.05 < SG < 0.1 in the proteomic tree, respectively (Fig. 3). In addition, genome alignment further revealed that Pan1 resembles Pam3 in several ORFs involved in nucleotide metabolism, and DNA replication and packing, despite most ORFs in the two genomes are largely reorganized (Additional file 1: Fig. S3A). In contrast, the two short-tailed cyanophages Pan3 and Pam1 are strongly collinear in a couple of ORFs that encode the structural proteins and DNA-binding proteins (Additional file 1: Fig. S3B). Altogether, despite infecting a same host *Pseudanabaena* sp. Chao 1811, the cyanophages Pan1~Pan5 are evolutionarily distinct from each other.

### Pan1 resembles a bacteriophage that infects α-proteobacteria

**Fig. 4:**
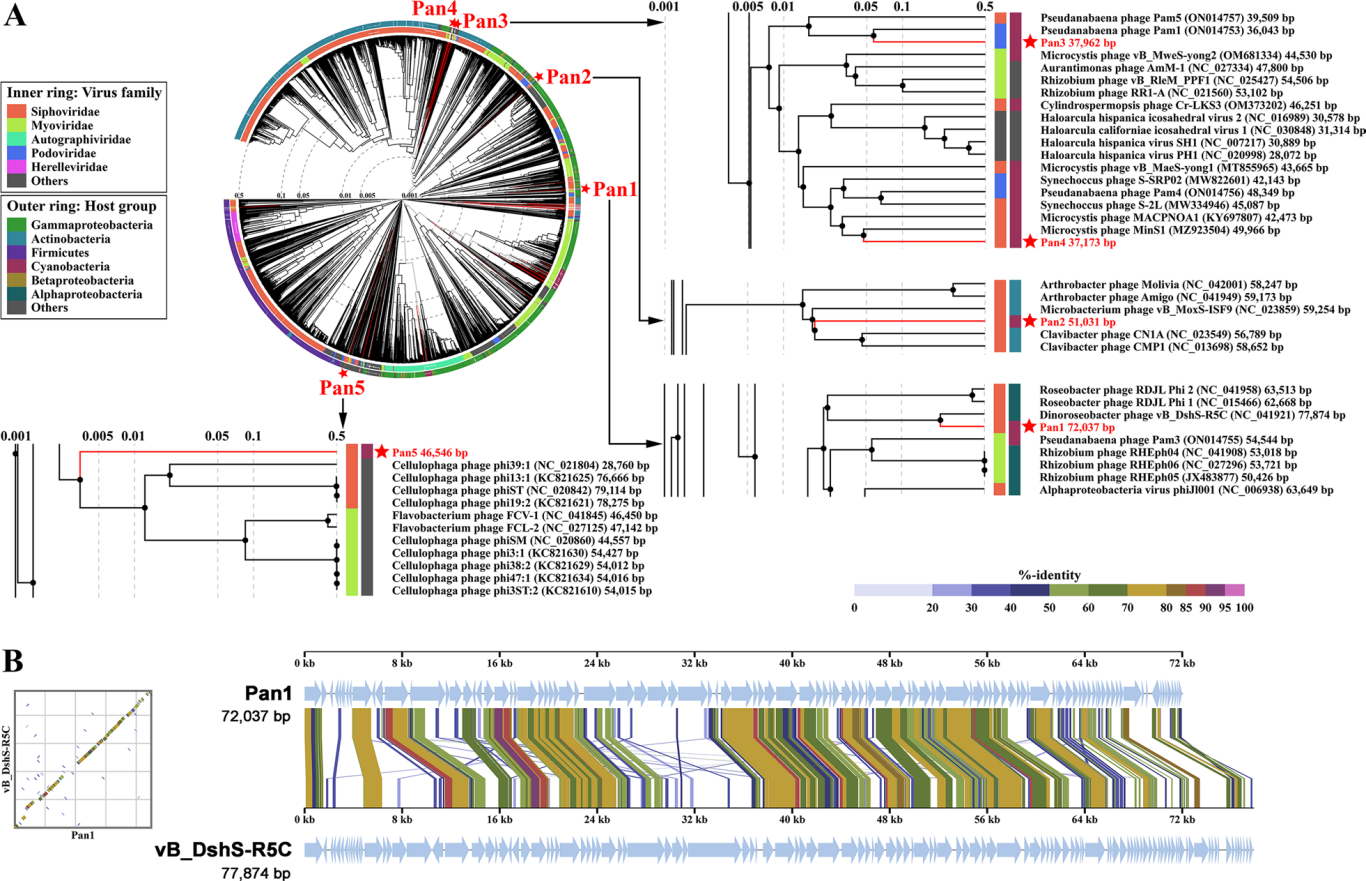
The phylogenetic analyses of Pan1~Pan5. **A** The proteomic tree of cyanophages Pan1~Pan5 against the genomes of 4918 dsDNA bacteriophages. The tree was generated by the ViPTree server [34]. Branch lengths were logarithmically scaled from the root of the entire proteomic tree. The inner ring represents the virus family, whereas the outer ring displays the host group. The red stars indicate the cyanophages Pan1~Pan5. **B** Whole-genome alignment of cyanophage Pan1 against bacteriophage vB_DshS-R5C. The alignment was performed with the software ViPTree [34]. The tBLASTx alignments are shown by lines in color between the two genomes, and the color scale at the top right represents the tBLASTx percent identity

A much global analysis with a proteomic tree against the genomes of 4,923 dsDNA bacteriophages using ViPTree software [34] showed that the cyanophages Pan1~Pan5 are not grouped into the same branch or adjacent branches, but are discretely distributed (Fig. 4A), suggesting their distinct evolutionary origins. According to the SG values, Pan1, Pan2 and Pan5 are respectively clustered in a branch together with bacteriophages to some extent (Fig. 4A). In contrast, Pan3 and Pan4 remain in the branches of cyanophages despite thousands of bacteriophages input to the proteomic tree, which are grouped with cyanophages Pam1 and MinS1 of the same tail morphology, respectively (Fig. 4A).

Different from the other four cyanophages, Pan1 shares a very close evolutionary origin (0.1 < SG < 0.5) with vB_DshS-R5C, a strain of siphophage that infects α-proteobacteria *D. shibae* DFL 12 isolated from the South China Sea [50]. Genome comparison revealed that Pan1 possesses a sequence identity of 72.02% over 53% of the vB_DshS-R5C genome. Moreover, whole-genome alignment showed that these two phages have plenty of collinear regions, beyond a couple of rearrangements in the genomes (Fig. 4B). Pan1 shares a homology of 60~90% in most ORFs with vB_DshS-R5C, such as the coding regions of several structural proteins (coat protein, tail protein, tail terminator, etc.), in addition to TerL and DNA polymerase III subunit (Fig. 4B). Altogether, Pan1 indeed resembles the bacteriophage vB_DshS-R5C infecting α-proteobacteria at the genomic level.

### Evolutionary traces of phage-mediated gene transfer between cyanobacteria and α-proteobacteria

**Fig. 5:**
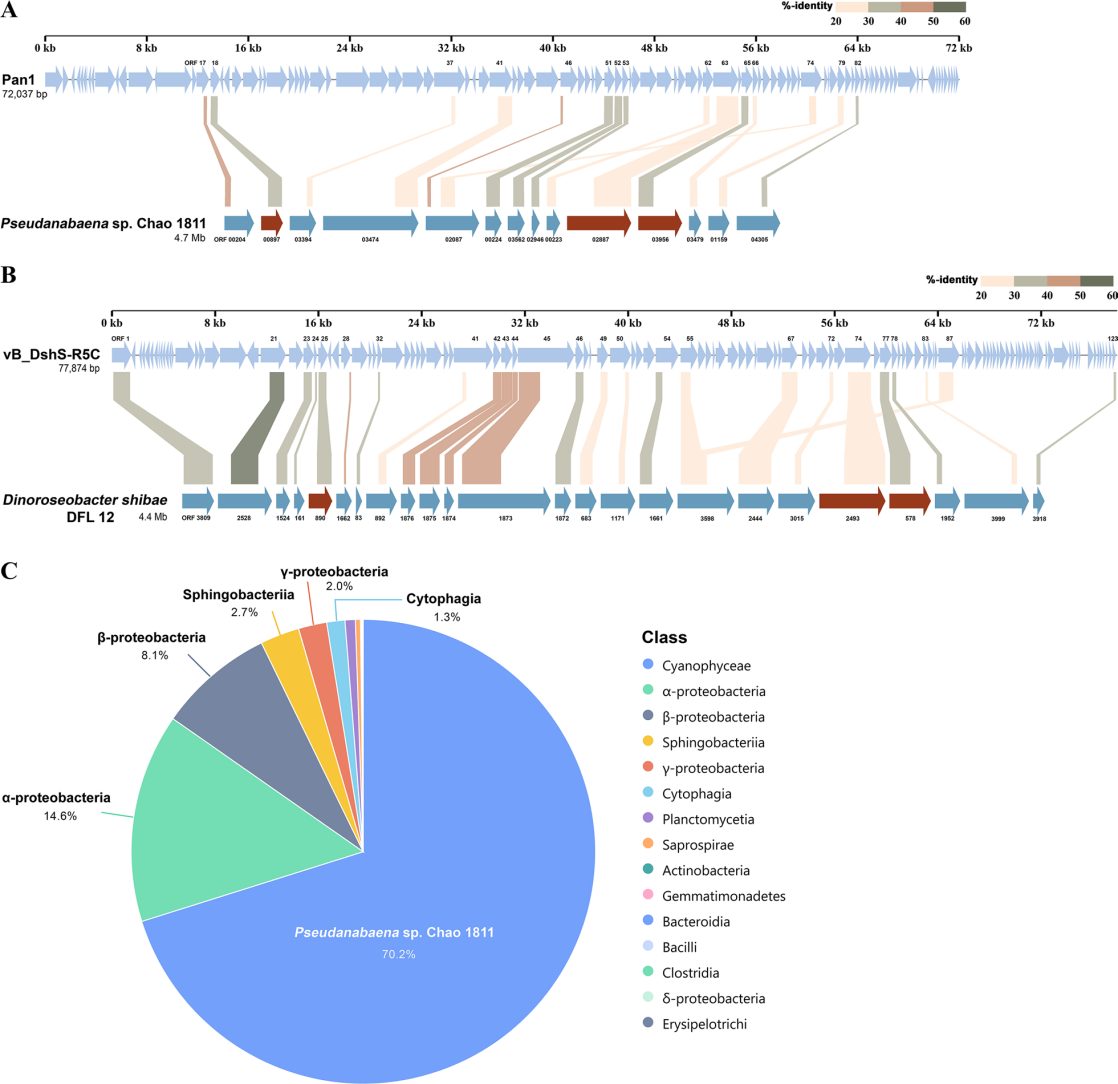
Evolutionary traces of inter-phylum gene transfer between cyanobacteria and *α*-proteobacteria. **A–B** BLASTp [36] analyses of Pan1 against host *Pseudanabaena* sp. Chao 1811 and vB_DshS-R5C against host *D. shibae* DFL 12, respectively. The threshold of the e-value is 0.05. The width of the lines represents the matching coverage of two proteins. The arrows in red highlight the homologous proteins shared by *Pseudanabaena* sp. Chao 1811 and *D. shibae* DFL 12. **C** The bacterial abundance of the cultured *Pseudanabaena* sp. Chao 1811 based on the reads of 16S rRNA sequencing

To rapidly adapt to the ever-changing environments during evolution, the phage could obtain genes from one host and pass them on to another via HGT [7, 51]. To screen the potential recombinations between the phage and its host, BLASTp [36] was applied to find the homologous proteins. In the range of 20~60% primary sequence identity, Pan1 shares 14 homologous proteins with *Pseudanabaena* sp. Chao 1811 (Fig. 5A), whereas vB_DshS-R5C has 24 homologous proteins with *D. shibae* DFL 12 (Fig. 5B). In detail, Pan1 resembles its host in three queuosine-related proteins (QueF, GCH and QueC), helicase, nuclease, and exonuclease, whereas vB_DshS-R5C is similar to its host in four GTA-like proteins, large terminase subunit, helicase and nuclease (Additional file 1: Table S4). Notably, three proteins PhoH, DNA polymerase I and DNA polymerase III subunit γ/τ are shared by the two phages and their hosts, at a sequence identity of 43%, 36% and 21% between *Pseudanabaena* sp. Chao 1811 and *D. shibae* DFL12, respectively. The phosphate starvation-inducible *phoH* is a well-known host-derived auxiliary metabolic gene [52, 53], which could be an indicator of HGT between phages and the host bacteria. Phylogenetic analyses showed that most PhoH proteins from cyanobacteria and *α*-proteobacteria were separately grouped; however, a couple of PhoH proteins from *α*-proteobacteria were grouped in the branch of cyanobacteria (Additional file 1: Fig. S4), indicating the exchange of *phoH* genes between the two phyla.

In fact, cyanobacteria and *α*-proteobacteria are abundant in various waterbodies [54–57]. A revisit of the 16S rRNA sequencing data of the *Pseudanabaena* sp. Chao 1811, which was purified via three rounds of single-colony inoculation on agar plates and subsequently amplified in the liquid medium, indicated that besides 70.1% *Pseudanabaena* sp. Chao 1811, *α*-proteobacteria accounts to 14.6% as the major contaminated species (Fig. 5C). It suggested that *Pseudanabaena* sp. Chao 1811 indeed prefers co-existing with *α*-proteobacteria in a same community, which provides the possibility for the inter-phylum gene transfer. Moreover, in the whole-genome sequencing data of liquid-medium amplified *Pseudanabaena* sp. Chao 1811, besides the reads constituting its own complete genome, a contig of *α*-proteobacterial sequences in length of 3,135,941 bp could be assembled as well. Sequence analysis showed that it also encodes an auxiliary metabolic protein PhoH (ORF2026), which shares a sequence identity of 26.87% and 40.60% to that of Pan1 and *Pseudanabaena* sp. Chao 1811, respectively. Notably, *Pseudanabaena* sp. Chao 1811 encodes a number of recombinases, integrases in addition to > 160 transposases, which might enable a high frequency of gene recombination.

As we known, inter-phylum gene transfer is usually mediated by a gene vector. For example, the gene transfer agent RcGTA, which was first identified in the *α*-proteobacterium *Rhodobacter capsulatus* that could mediate the gene transfer [58], also adopts a phage-like structure [59]. Our cyanophage Pan1 also encodes three RcGTA-like proteins gp37, gp96 and gp101, further implying the traces of gene transfer from *α*-proteobacteria. Besides, the above mentioned *phoH* gene that exists in the two phages (Pan1 and vB_DshS-R5C) and their hosts (cyanobacteria and *α*-proteobacteria), provides direct evidence of phage-mediated inter-phylum gene transfer.

## Discussion

Compared to extensive investigations on marine cyanophages, only 32 freshwater cyanophages have been experimentally isolated and applied to whole-genome sequencing (Additional file 1: Table S5). Here we isolated and sequenced five freshwater cyanophages Pan1~Pan5 with two types of tail morphology (Figs. 1 and 2), which infect the host *Pseudanabaena* sp. Chao 1811. Comparative genomic and proteomic analyses showed that Pan1~Pan5 could be clustered with other freshwater cyanophages, but are located in different branches (Fig. 3). The five cyanophages characterized in the present study, together with our previously reported Pam1~Pam5 [24], significantly enrich the library of reference freshwater cyanophages.

Upon the rapid development of genomics, metagenomics, and metabolomics, a growing number of studies have been focused on the interrelation between cyanobacteria and the attached bacteria, including the community succession dynamics, co-metabolic pathways, material exchanges and signal transmission [60–62]. In this symbiosis system, cyanobacteria could provide energy for their neighboring heterotrophic bacteria, whereas the attached bacteria in turn supply nitrogen and phosphorous for cyanobacteria [63, 64]. The cyanobacteria *Synechococcus* and heterotrophic bacteria could survive permanently without the supply of external nutrients, after changing their relationship from antagonism to mutualism during long-term cocultivation [62]. Using the single-colony sequencing, a microbe-by-microbiome phylosymbiosis between the cyanobacteria *Microcystis* and its attached bacteria was revealed, and possible HGT events were inferred between *Microcystis* and a couple of its microbiome members in a similar geographical niche [57]. Genome assembly and metabolic pathway analyses of cyanobacterial aggregates showed that cyanobacteria and the attached bacteria usually form functional links via nitrogen- and/or phosphorus-cycling co-pathways, suggesting the exchange and sharing of auxiliary metabolic genes [65, 66]. These studies also support our hypothesis that HGT events likely occur between cyanobacteria and the attached *α*-proteobacteria, which usually live in a same community in Lake Chaohu.

HGT, a pillar of bacterial evolution, can occur via various mechanisms, which transfers multiple genes in bulk or only short DNA segments, along with mutation, genetic drift, selection and dispersal [5]. It triggers diverse consequences to the acceptor bacteria, ranging from altering phylogenetic signals among genes to fueling adaptive evolution to new niches via a few recombination events [67, 68]. The similarities between Pan1 and Pam3, as well as Pan3 and Pam1 at the genomic level (Additional file 1: Fig. S3), suggested a series of evolutionary traces of HGT among phages that infect a similar host of *Pseudanabaena*. Different from this phage-mediated HGT constrained in a specific host range, it is extremely difficult to exchange genes between cyanophage Pan1 and bacteriophage vB_DshS-R5C, which respectively infect the hosts in two completely different phyla.

Here we propose that the phage obtained the auxiliary metabolic gene, for example *phoH*, from its host after infection; and the progeny phage is released to the waterbodies. In such an open aquatic environment, the DNA fragments released from the phage genome, including the DNA fragment encoding PhoH, might be naturally absorbed by a competent bacterium/cyanobacterium. During replication of phages in the transformed host, this DNA fragment could be further integrated into the genome of the progeny phages, and is passed on upon the next round of infection. Meanwhile, the DNA fragment could also be inserted into the genome of the transformed bacterium/cyanobacterium, accompanying with the lysogenesis of the infected phage or simply via DNA recombination. However, more investigations are needed to prove this hypothesis of phage-mediated inter-phylum gene transfer.

## Conclusion

In this study, we isolated and identified five freshwater cyanophages Pan1~Pan5 that infect *Pseudanabaena* sp. Chao 1811, the comparative genomic analyses of which against other cyanophages/bacteriophages, enabled us to better understand the evolutionary diversity of freshwater cyanophages. Moreover, via phylogenetic analyses, a series of evolutionary traces of gene transfer were characterized among various phages, or between the hosts cyanobacteria and the attached *α*-proteobacteria. It indicated a phage-mediated inter-phylum horizontal gene transfer, which would endow bacteria novel features to adapt to the fluctuating environments during evolution. However, more studies are needed to prove this inter-phylum gene transfer.

## Supplementary Information

**Additional file 1.** Supplementary figures and tables.

## Data Availability

The genomes of cyanophages Pan1 ~ Pan5 have been deposited in the GenBank database with the accession numbers ON968452 ~ ON968456, respectively. The genome of *Pseudanabaena* sp. Chao 1811 has been deposited into the GenBank database under the accession number CP101416. The raw 16S rRNA sequencing data has been deposited into the Sequence Read Archive (SRA) database under the Bioproject number PRJNA857886. All data generated or analyzed during this study are included in this published article and its supplementary information files.
