# Supplementary material for: Phylogenomics of five Pseudanabaena cyanophages and evolutionary traces of horizontal gene transfer
Source: Environ Microbiome. 2023 Jan 13;18:3. doi: 10.1186/s40793-023-00461-5 (PMC9837993; doi:10.1186/s40793-023-00461-5)
Supplement: Supplementary file 1 — Additional file 1. Supplementary figures and tables. [file 40793_2023_461_MOESM1_ESM.pdf]

**Supporting information**

**Supplementary figures and figure legends**

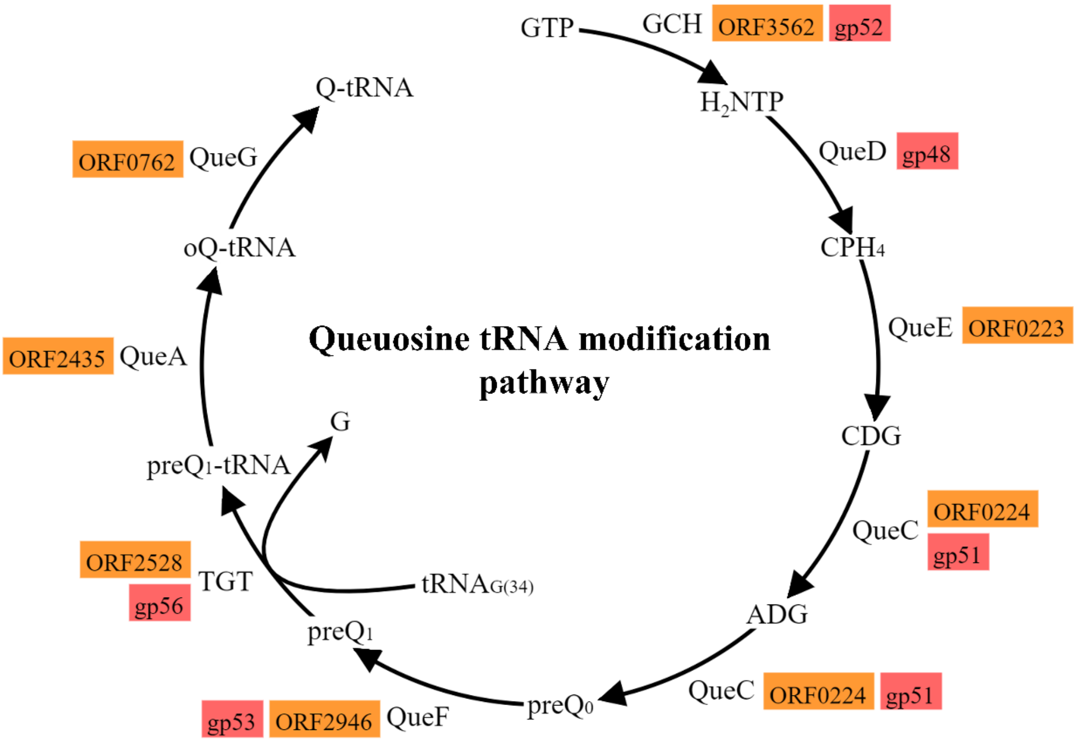


**Fig. S1** A diagram of queuosine tRNA modification pathway. The red boxes indicate the enzymes encoded by Pan1, whereas the orange boxes represent the enzymes from host *Pseudanabaena* sp. Chao 1811. QueD, encoded by Pan1 as a salvage synthase, together with other eight enzymes of *Pseudanabaena* sp. Chao 1811, constitutes a complete pathway for queuosine tRNA modification. GCH: GTP cyclohydrolase I; QueD: 6-carboxytetrahydropterin synthase; QueC: 7-cyano-7-deazaguanine synthase; QueE: 7-carboxy-7-deazaguanine synthase; QueF: NADPH-dependent 7-cyano-7-deazaguanine reductase; TGT: tRNA guanine(34) transglycosylase; QueA: tRNA $preQ_1$(34) S-adenosylmethionine ribosyltransferase-isomerase; QueG: tRNA

epoxyqueuosine(34) reductase; GTP: Guanosine triphosphate; $H_2NTP$: 7,8-dihydroneopterin-3′-triphosphate; $CPH_4$: 6-pyruvoyl-5,6,7,8-tetrahydropterin ; CDG: 7-carboxy-7-deazaguanine ; ADG: 7-amido-7-deazaguanine; $preQ_0$: 7-cyano-7-deazaguanine; $preQ_1$: 7-aminomethyl-7-deazaguanine; $preQ_1$-tRNA: $preQ_1$ at the position 34 of tRNA; oQ-tRNA: Epoxyqueuosine at the position 34 of tRNA; Q-tRNA: Queuosine at the position 34 of tRNA; $tRNA_{G(34)}$: Guanine at the position 34 of tRNA; G: Guanine; tRNA: transfer RNA.

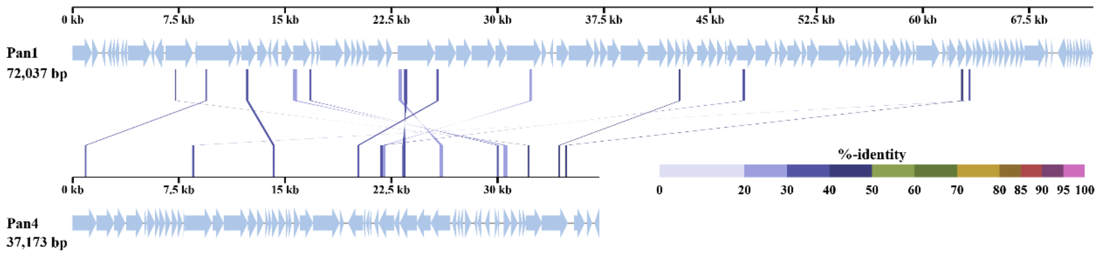


**Fig. S2** Whole-genome alignment of cyanophage Pan1 against Pan4. The alignment was performed with the software ViPTree. The tBLASTx alignments are showed by lines in color between the two genomes, and the color scale at the bottom right represents the tBLASTx percent identity.

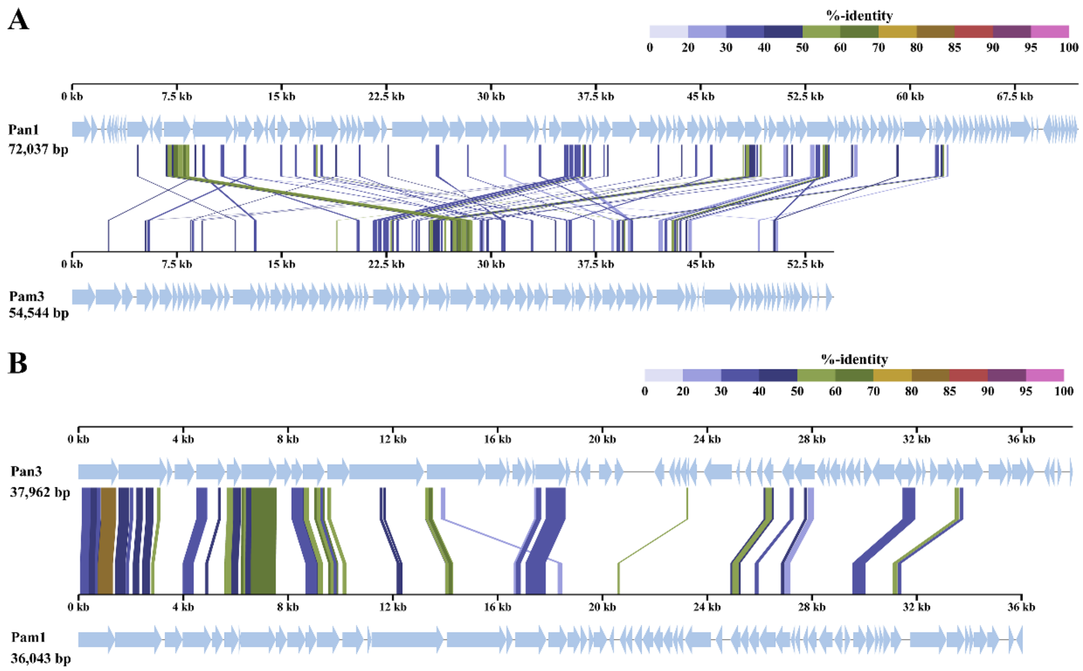


**Fig. S3** Whole-genome alignments of **A** Pan1 against Pam3 and **B** Pan3 against Pam1, respectively. The alignments were performed by ViPTree. The tBLASTx alignments are showed by lines in color between the two genomes, and the color scale at the top right represents the tBLASTx percent identity.

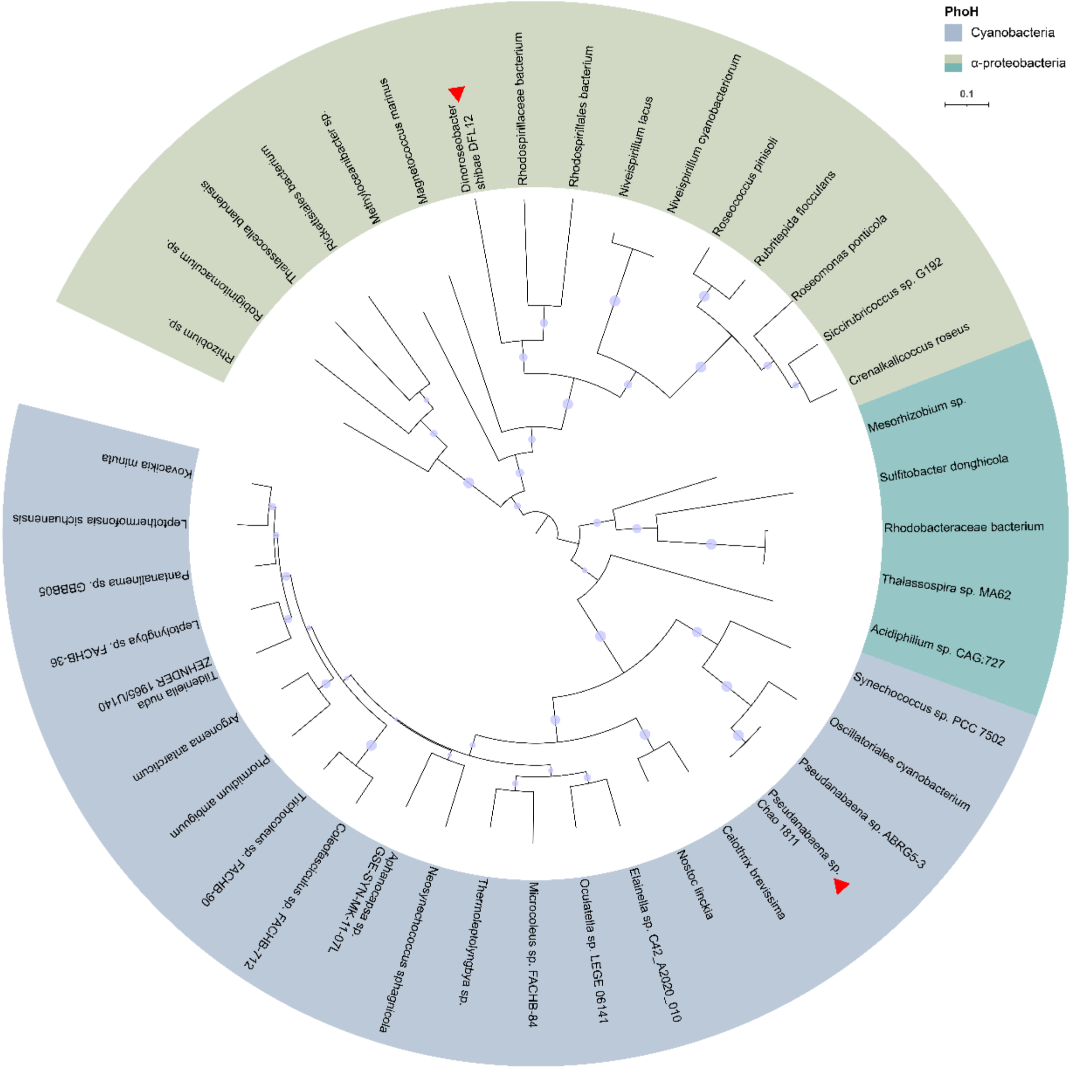


**Fig. S4** Phylogenetic analyses of 42 PhoH proteins from different species of cyanobacteria (blue) and α-proteobacteria (green and aquamarine) via MEGA 11 software. The host of cyanophage Pan1, *Pseudanabaena* sp. Chao 1811, and the host of bacteriophage vB_DshS-R5C, *D. shibae* DFL 12, are indicated by red triangles, respectively. The size of lavender circles at the branches represents the bootstrap values, of which larger value means higher confidence. A couple of PhoH proteins from α-proteobacteria, colored with aquamarine frame, are grouped in the branch of cyanobacteria, suggesting the exchange of *phoH* genes between the two phyla.

## Supplementary tables

**Table S1.** The parameters of cyanophages Pan1~Pan5 that infect *Pseudanabaena* sp. Chao 1811.

| Phage | Morphology | Head size (nm) | Tail length (nm) | Genome size (kb) | G+C content (%) | Volume ($nm^3$) | Genome size per volume ($bp/nm^3$) |
|---|---|---|---|---|---|---|---|
| Pan1 | *Siphoviridae* | 94/58 | 144 | 72.04 | 63 | 100240.47 | 0.72 |
| Pan2 | *Siphoviridae* | 70 | 210 | 51.03 | 59 | 108731.00 | 0.47 |
| Pan3 | *Podoviridae* | 67 | - | 37.96 | 61 | 95341.87 | 0.40 |
| Pan4 | *Siphoviridae* | 62 | 100 | 37.17 | 64 | 75549.98 | 0.49 |
| Pan5 | *Siphoviridae* | 67 | 149 | 46.55 | 44 | 95341.87 | 0.49 |

**Table S2A.** Predicted ORFs with known functions of cyanophage Pan1.

| ORF | Start (bp) | Stop (bp) | Strand[1] | Length (aa) | E-value[2] | Predicted protein |
|---|---|---|---|---|---|---|
| 1 | 1 | 1389 | + | 462 | 0 | Large terminase subunit |
| 2 | 1389 | 1829 | + | 146 | 5.40E-21 | Kinase-like protein |
| 10 | 3932 | 5521 | + | 529 | 2.70E-27 | Portal |
| 13 | 6574 | 8508 | + | 644 | 0 | Ribonucleotide reductase |
| 15 | 8766 | 11549 | + | 927 | 6.30E-16 | Head morphogenesis protein |
| 17 | 11913 | 12923 | + | 336 | 4.30E-29 | N-acetylmuramidase |
| 18 | 13019 | 13765 | + | 248 | 1.00E-107 | Phosphate starvation-inducible protein |
| 21 | 14726 | 15499 | + | 257 | 1.80E-09 | Minor structural protein |
| 22 | 15580 | 16809 | + | 409 | 0 | P22-like coat protein |
| 25 | 17462 | 19093 | + | 543 | 0 | DNA transfer protein |
| 26 | 19187 | 19672 | + | 161 | 2.00E-80 | Minor tail protein |
| 27 | 19669 | 20040 | + | 123 | 2.20E-05 | Tail attachment protein |
| 28 | 20040 | 20453 | + | 137 | 2.00E-65 | Virion structural protein |
| 29 | 20450 | 20860 | + | 136 | 2.00E-59 | Structural protein |
| 30 | 20880 | 22082 | + | 400 | 3.10E-16 | Tail tube protein |
| 31 | 22160 | 22552 | + | 130 | 4.00E-49 | Pre-tape measure chaperone protein |
| 32 | 22928 | 25558 | + | 876 | 2.00E-128 | Tail tape measure protein |
| 33 | 25558 | 27051 | + | 497 | 1.60E-04 | Distal tail protein |
| 37 | 30638 | 33082 | + | 814 | 3.60E-66 | GTA-like baseplate hub protein |
| 40 | 34161 | 35009 | + | 282 | 3.00E-133 | Thymidylate synthase |
| 41 | 35012 | 36784 | + | 590 | 0 | Superfamily II DNA/RNA helicase |
| 43 | 37180 | 37722 | + | 180 | 3.00E-64 | Heat shock protein |
| 45 | 38688 | 40445 | + | 585 | 0 | Primase |
| 46 | 40432 | 41952 | + | 506 | 0 | ATP-dependent DNA helicase |
| 47 | 41946 | 42521 | + | 191 | 8.00E-61 | Phosphoglycolate phosphatase |
| 48 | 42523 | 42969 | + | 148 | 3.00E-73 | 6-carboxytetrahydropterin synthase |
| 51 | 44054 | 44887 | + | 277 | 1.00E-158 | 7-cyano-7-deazaguanine synthase |
| 52 | 44887 | 45465 | + | 192 | 2.00E-113 | Putative GTP cyclohydrolase |
| 53 | 45475 | 45948 | + | 157 | 1.00E-53 | QueF |
| 55 | 46226 | 46876 | + | 216 | 4.00E-55 | YkgJ family cysteine cluster protein |
| 56 | 46880 | 48181 | + | 433 | 0 | Queuosine tRNA-ribosyltransferase |
| 57 | 48181 | 49392 | + | 403 | 0 | Exodeoxyribonuclease-5 |
| 59 | 49809 | 50435 | + | 208 | 4.00E-110 | DNA polymerase III subunit ε |
| 60 | 50447 | 51430 | + | 327 | 1.40E-40 | DNA polymerase III subunit β |
| 62 | 51812 | 52630 | + | 272 | 3.00E-143 | Radical SAM protein |
| 63 | 52630 | 54609 | + | 659 | 0 | DNA polymerase I |
| 65 | 54817 | 55746 | + | 309 | 2.00E-162 | DNA polymerase III subunit γ/τ |
| 68 | 56524 | 57114 | + | 196 | 2.00E-90 | Transcription elongation protein |
| 74 | 59549 | 61189 | + | 546 | 0 | ATP-dependent DNA helicase |
| 78 | 61788 | 62417 | + | 209 | 1.70E-38 | Nucleotide modification associated domain 5 |

| | | | | | | |
|---|---|---|---|---|---|---|
| 79 | 62429 | 63040 | + | 203 | 2.00E-25 | Exonuclease |
| 82 | 63784 | 64230 | + | 148 | 1.30E-16 | Nuclease |
| 96 | 69956 | 70201 | + | 81 | 1.70E-10 | GTA-like holin |
| 101 | 71139 | 71375 | + | 78 | 1.30E-22 | GTA-like head spike base protein |

ORF number, gene position and protein size are noted for each predicted protein-coding gene.

[1]represented the gene orientation, + is forward strand, and - is reverse strand.

[2]predicted by BLASTp or HHpred.

**Table S2B.** Predicted ORFs with known functions of cyanophage Pan2.

| ORF | Start (bp) | Stop (bp) | Strand[1] | Length (aa) | E-value[2] | Predicted protein |
|---|---|---|---|---|---|---|
| 1 | 1 | 1506 | + | 501 | 9.00E-110 | Large terminase subunit |
| 2 | 1525 | 3087 | + | 520 | 1.50E-26 | Portal |
| 3 | 3136 | 4101 | + | 321 | 6.20E-12 | Prohead protease |
| 5 | 4457 | 5572 | + | 371 | 4.70E-24 | Mu-like prophage major head subunit |
| 9 | 7044 | 7253 | + | 69 | 5.00E-23 | HNH endonuclease |
| 10 | 7262 | 7975 | + | 161 | 3.00E-06 | T9SS type A sorting domain-containing protein |
| 11 | 7975 | 8400 | + | 141 | 7.90E-08 | Putative tail-component protein |
| 12 | 8397 | 8828 | + | 143 | 7.10E-07 | GTA-like adaptor protein |
| 15 | 9608 | 10360 | + | 250 | 4.00E-23 | Capsid protein |
| 18 | 11540 | 15091 | + | 1183 | 0 | Tail tape measure protein |
| 23 | 19154 | 19885 | + | 243 | 2.30E-15 | D-alanyl-D-alanine carboxypeptidase |
| 25 | 20246 | 20971 | - | 241 | 7.00E-105 | Ribonuclease H-like domain-containing protein |
| 29 | 21622 | 21984 | - | 120 | 2.00E-36 | HNH endonuclease |
| 30 | 22052 | 22519 | - | 155 | 2.00E-12 | RuvC resolvase |
| 39 | 24545 | 26770 | - | 741 | 9.90E-14 | Primase |
| 41 | 27439 | 27816 | - | 125 | 6.00E-24 | Single-stranded DNA-binding protein |
| 52 | 32396 | 32884 | - | 162 | 2.00E-71 | Adenine methyltransferase |
| 53 | 32902 | 34290 | - | 462 | 3.00E-95 | HeLICc domain containing protein |
| 71 | 38799 | 39332 | - | 177 | 6.10E-23 | HNH endonuclease |
| 73 | 39878 | 40540 | - | 220 | 3.60E-18 | DNA polymerase III subunit β |
| 90 | 46643 | 47191 | + | 182 | 1.00E-11 | Endonuclease VII domain-containing protein |

[1]represented the gene orientation, + is forward strand and - is reverse strand.

[2]predicted by BLASTp or HHpred.

**Table S2C.** Predicted ORFs with known functions of cyanophage Pan3.

| ORF | Start (bp) | Stop (bp) | Strand[1] | Length (aa) | E-value[2] | Predicted protein |
|---|---|---|---|---|---|---|
| 1 | 1 | 1524 | + | 507 | 0 | Large terminase subunit |
| 2 | 1521 | 3383 | + | 620 | 1.30E-44 | Portal |
| 5 | 4490 | 5599 | + | 369 | 0 | Major capsid protein |
| 6 | 5654 | 6214 | + | 186 | 3.30E-23 | Adaptor |
| 7 | 6214 | 7554 | + | 446 | 5.40E-47 | Phage stabilisation protein |
| 8 | 7551 | 8144 | + | 197 | | Needle* |
| 10 | 8565 | 9392 | + | 275 | | Ejection protein* |
| 12 | 9501 | 10343 | + | 280 | | Ejection protein* |
| 13 | 10345 | 13185 | + | 946 | | Ejection protein* |
| 14 | 13298 | 15535 | + | 745 | | Tail spike* |
| 20 | 17449 | 18615 | + | 388 | 1.00E-172 | LysR family transcriptional regulator |
| 25 | 19880 | 20374 | + | 164 | 1.20E-24 | Lysozyme |
| 29 | 22534 | 22755 | - | 73 | 1.10E-06 | YjhX_toxin |
| 34 | 23870 | 24940 | - | 356 | 7.20E-37 | Integrase |
| 39 | 26152 | 26523 | - | 123 | 1.00E-45 | Recombination protein NinB |
| 40 | 26845 | 27318 | - | 157 | 1.00E-66 | Single-stranded DNA-binding protein |
| 41 | 27318 | 28097 | - | 259 | 2.00E-71 | ERF family protein |
| 55 | 32804 | 33439 | + | 211 | 8.00E-78 | Site-specific DNA-methyltransferase |
| 56 | 33436 | 33786 | + | 116 | 7.00E-29 | VRR-NUC domain-containing protein |
| 65 | 37526 | 37960 | + | 146 | 3.00E-37 | Small terminase subunit |

[1]represented the gene orientation, + is forward strand and - is reverse strand.

[2]predicted by BLASTp or HHpred.

*identified by cryo-electron microscopy or predication of Alphafold2.

**Table S2D.** Predicted ORFs with known functions of cyanophage Pan4.

| ORF | Start (bp) | Stop (bp) | Strand[1] | Length (aa) | E-value[2] | Predicted protein |
|---|---|---|---|---|---|---|
| 1 | 1 | 1698 | + | 565 | 0 | Large terminase subunit |
| 2 | 1695 | 2939 | + | 414 | 0 | Portal |
| 3 | 2936 | 3715 | + | 259 | 1.00E-82 | Clp protease |
| 4 | 3776 | 4987 | + | 403 | 0 | Major capsid protein |
| 6 | 5268 | 5813 | + | 181 | 1.10E-26 | GTA-like adaptor protein |
| 7 | 5810 | 6139 | + | 109 | 9.00E-21 | Putative head-tail adaptor |
| 8 | 6136 | 6531 | + | 131 | 5.50E-11 | Tail-component protein |
| 9 | 6531 | 6938 | + | 135 | 5.80E-19 | GTA-like tail terminator protein |
| 10 | 7022 | 7417 | + | 131 | 2.20E-19 | Phage major tail protein |
| 11 | 7417 | 7758 | + | 113 | 3.70E-13 | GTA-like tail tube protein |
| 15 | 10653 | 12368 | + | 571 | 1.30E-08 | Putative phage tail protein |
| 21 | 14530 | 15018 | + | 162 | 2.80E-16 | M15 family metallopeptidase |
| 28 | 19437 | 20489 | - | 350 | 2.00E-180 | Integrase |
| 29 | 20486 | 20692 | - | 68 | 6.80E-08 | Tor inhibition protein |
| 35 | 23070 | 24284 | - | 404 | 0 | DNA cytosine methyltransferase |
| 36 | 24281 | 25288 | - | 335 | 0 | DNA methylase |
| 37 | 25267 | 26649 | - | 460 | 0 | Helicase |
| 44 | 29088 | 29423 | - | 111 | 9.10E-38 | XRE family transcriptional regulator |
| 51 | 32008 | 33129 | + | 373 | 1.80E-28 | Primase |
| 52 | 33126 | 34952 | + | 608 | 0 | Putative DNA primase/helicase |
| 54 | 36279 | 36653 | + | 124 | 2.00E-46 | HNH endonuclease |
| 55 | 36635 | 37171 | + | 179 | 1.60E-05 | Small terminase subunit |

[1]represented the gene orientation, + is forward strand and - is reverse strand.

[2]predicted by BLASTp or HHpred.

**Table S2E.** Predicted ORFs with known functions of cyanophage Pan5.

| ORF | Start (bp) | Stop (bp) | Strand[1] | Length (aa) | E-value[2] | Predicted protein |
|---|---|---|---|---|---|---|
| 1 | 1 | 1440 | + | 479 | 9.00E-95 | Large terminase subunit |
| 2 | 1519 | 3186 | + | 555 | 3.90E-15 | Portal |
| 6 | 4875 | 5918 | + | 347 | | Major capsid protein* |
| 7 | 5945 | 6346 | + | 133 | | Cement* |
| 8 | 6346 | 7368 | + | 340 | | Adaptor* |
| 9 | 7362 | 7916 | + | 184 | | Tail terminator protein* |
| 12 | 9105 | 9539 | + | 144 | 1.50E-11 | Tail completion protein |
| 17 | 16609 | 17994 | + | 461 | 4.30E-08 | Receptor-binding domain of short tail fibre protein |
| 19 | 20965 | 22086 | + | 373 | 3.00E-33 | Heparin lyase I family protein |
| 25 | 24468 | 24923 | + | 151 | 1.00E-45 | Peptidoglycan L-alanyl-D-glutamate endopeptidase precursor |
| 29 | 25957 | 26253 | + | 98 | 1.00E-17 | Helix-turn-helix transcriptional regulator |
| 37 | 28444 | 29127 | + | 227 | 9.70E-19 | RecT protein |
| 38 | 29117 | 29635 | + | 172 | 3.00E-33 | HNH endonuclease |
| 39 | 29632 | 30417 | + | 261 | 1.00E-59 | YqaJ viral recombinase family protein |
| 41 | 31598 | 31888 | + | 96 | 4.10E-07 | Omega transcriptional repressor |
| 44 | 32889 | 33284 | + | 131 | 3.20E-13 | HNH endonuclease |
| 47 | 34103 | 34834 | + | 243 | 8.00E-42 | HNH endonuclease |
| 49 | 35493 | 35873 | + | 126 | 8.00E-27 | HNH endonuclease |
| 55 | 38116 | 38460 | + | 114 | 2.50E-09 | Restriction endonuclease AbaSI |
| 56 | 38457 | 39329 | + | 290 | 7.00E-160 | DNA (cytosine-5-)-methyltransferase |
| 57 | 39341 | 41890 | + | 849 | 8.30E-35 | Restriction endonuclease ecoP15I, modification subunit |
| 59 | 42302 | 42550 | + | 82 | 2.90E-07 | Z-DNA-binding protein 1 |
| 60 | 42554 | 43183 | + | 209 | 2.00E-25 | HNH endonuclease |
| 61 | 43180 | 43560 | + | 126 | 2.50E-06 | YorP protein |
| 71 | 46105 | 46545 | + | 147 | 3.00E-48 | Small terminase subunit |

[1]represented the gene orientation, + is forward strand and - is reverse strand.

[2]predicted by BLASTp or HHpred.

*identified by cryo-electron microscopy or predication of Alphafold2.

**Table S3.** The queuosine tRNA modification genes in cyanophage Pan1 and its host *Pseudanabaena* sp. Chao 1811.

| Pan1 | Function | Chao 1811 | Coverage (%) | Identity (%) |
|---|---|---|---|---|
| gp48 | 6-carboxytetrahydropterin synthase (QueD) | - | - | - |
| gp51 | 7-cyano-7-deazaguanine synthase (QueC) | ORF0224 | 80 | 33.78 |
| gp52 | GTP cyclohydrolase (GCH) | ORF3562 | 88 | 31.36 |
| gp53 | NADPH-dependent 7-cyano-7-deazaguanine reductase (QueF) | ORF2946 | 78 | 39.02 |
| gp56 | Queuosine tRNA-ribosyltransferase (TGT) | ORF2528 | - | - |
| - | 7-carboxy-7-deazaguanine synthase (QueE) | ORF0223 | - | - |
| - | tRNA epoxy-queuosine (34) reductase (QueG) | ORF0762 | - | - |
| - | S-adenosylmethionine/tRNA ribosyltransferase-isomerase (QueA) | ORF2435 | - | - |

**Table S4A.** The homologous proteins of Pan1 with host *Pseudanabaena* sp. Chao 1811.

| Pan1 | Function | Start (aa) | Stop (aa) | Chao 1811 | Function | Start (aa) | Stop (aa) | Identity (%) | E-value* |
|---|---|---|---|---|---|---|---|---|---|
| ORF17 | N-acetylmuramidase | 199 | 279 | ORF0204 | Hypothetical protein | 4 | 89 | 43.02 | 9.03E-12 |
| ORF46 | ATP-dependent DNA helicase | 6 | 55 | ORF2087 | ATP-dependent DNA helicase | 23 | 73 | 41.18 | 7.03E-05 |
| ORF53 | QueF | 25 | 147 | ORF2946 | QueF | 7 | 123 | 39.02 | 1.05E-21 |
| ORF65 | DNA polymerase III subunit $\gamma/\tau$ | 1 | 194 | ORF3956 | DNA polymerase III subunit $\gamma/\tau$ | 1 | 219 | 38.81 | 2.74E-39 |
| ORF18 | PhoH | 14 | 229 | ORF0897 | PhoH | 105 | 318 | 37.04 | 9.03E-37 |
| ORF51 | QueC | 2 | 223 | ORF0224 | QueC | 13 | 227 | 33.78 | 8.49E-29 |
| ORF82 | Nuclease | 12 | 86 | ORF4305 | Hypothetical protein | 375 | 455 | 32.93 | 1.80E-02 |
| ORF52 | GTP cyclohydrolase | 19 | 187 | ORF3562 | GTP cyclohydrolase | 81 | 248 | 31.36 | 4.32E-26 |
| ORF66 | Hypothetical protein | 3 | 108 | ORF3479 | tRNA-specific adenosine deaminase | 15 | 131 | 29.66 | 4.14E-08 |
| ORF62 | Radical SAM protein | 18 | 169 | ORF0223 | QueE | 9 | 145 | 28.11 | 3.56E-12 |
| ORF74 | ATP-dependent DNA helicase | 211 | 382 | ORF2087 | ATP-dependent DNA helicase | 228 | 437 | 28.04 | 1.48E-08 |
| ORF63 | DNA polymerase I | 73 | 658 | ORF2887 | DNA polymerase I | 395 | 951 | 27.75 | 1.31E-47 |
| ORF37 | GTA-like baseplate hub protein | 450 | 543 | ORF3394 | Protein-glutamate methylesterase/protein-glutamine glutaminase | 256 | 349 | 27.37 | 2.00E-02 |
| ORF79 | Exonuclease | 7 | 152 | ORF1159 | Ribosomal protein L11 methyltransferase | 158 | 280 | 23.29 | 4.50E-02 |
| ORF41 | Superfamily II DNA/RNA helicase | 223 | 588 | ORF3474 | RNA polymerase-associated protein | 1066 | 1404 | 22.56 | 2.37E-13 |

* predicted by BLASTp, the threshold of e-value is 0.05.

**Table S4B.** The homologous proteins of vB_DshS-R5C with host *D. shibae* DFL 12.

| R5C | Accession number | Function | Start (aa) | Stop (aa) | DFL 12 | Accession number | Function | Start (aa) | Stop (aa) | Identity (%) | E-value* |
|---|---|---|---|---|---|---|---|---|---|---|---|
| ORF21 | YP_009600167 | Ribonucleotide reductase | 231 | 611 | ORF2528 | WP_012179610 | Ribonucleoside-diphosphate reductase | 187 | 574 | 50.38 | 1.53E-117 |
| ORF42 | YP_009600188 | GTA-like protein | 1 | 181 | ORF1876 | WP_012178834 | DUF2460 domain-containing protein | 26 | 210 | 47.57 | 2.86E-64 |
| ORF44 | YP_009600190 | GTA-like protein | 3 | 134 | ORF1874 | WP_012178832 | Peptidase | 7 | 143 | 45.99 | 1.50E-34 |
| ORF28 | YP_009600174 | Hypothetical protein | 226 | 254 | ORF1662 | WP_012178582 | Hypothetical protein | 117 | 145 | 44.83 | 3.20E-02 |
| ORF45 | YP_009600191 | GTA-like protein | 26 | 577 | ORF1873 | WP_012178831 | Phage tail family protein | 54 | 605 | 44.54 | 3.62E-131 |
| ORF43 | YP_009600189 | GTA-like protein | 7 | 291 | ORF1875 | WP_012178833 | DUF2163 domain-containing protein | 7 | 294 | 40.14 | 2.68E-72 |
| ORF1 | YP_009600147 | Large terminase subunit | 37 | 490 | ORF3809 | WP_083768379 | Terminase family protein | 18 | 444 | 39.32 | 1.31E-87 |
| ORF25 | YP_009600171 | PhoH | 12 | 227 | ORF0890 | WP_012177689 | PhoH | 120 | 333 | 37.62 | 1.65E-43 |
| ORF54 | YP_009600200 | Primase | 3 | 173 | ORF1661 | WP_012178579 | Primase | 8 | 176 | 36.57 | 1.52E-20 |
| ORF78 | YP_009600224 | Deoxycytidylate deaminase | 22 | 114 | ORF1952 | WP_012178923 | RibD protein | 24 | 104 | 34.34 | 4.30E-02 |
| ORF46 | YP_009600192 | Ribonuclease III | 3 | 235 | ORF1872 | WP_012178830 | DUF2793 domain-containing protein | 4 | 232 | 33.62 | 4.46E-40 |
| ORF24 | YP_009600170 | Hypothetical protein | 37 | 75 | ORF0161 | WP_012176819 | RidA family protein | 4 | 41 | 33.33 | 1.50E-02 |
| ORF32 | YP_009600178 | Hypothetical protein | 23 | 84 | ORF0083 | WP_012176722 | DUF1330 domain-containing protein | 17 | 73 | 33.33 | 1.70E-02 |
| ORF77 | YP_009600223 | DNA polymerase III subunit γ/τ | 3 | 245 | ORF0578 | WP_012177328 | DNA polymerase III subunit γ/τ | 10 | 297 | 31.25 | 1.66E-29 |
| ORF123 | YP_009600146 | Hypothetical protein | 40 | 99 | ORF3918 | WP_157865346 | YbjQ family protein | 55 | 113 | 31.15 | 7.00E-03 |
| ORF23 | YP_009600169 | Acetylmuramidase | 5 | 165 | ORF1524 | WP_012178429 | N-acetylmuramidase | 3 | 171 | 30.46 | 1.23E-13 |
| ORF41 | YP_009600187 | Tail tape measure protein | 226 | 319 | ORF0892 | WP_012177692 | tRNA-methylthiotransferase | 174 | 286 | 29.92 | 2.90E-02 |
| ORF72 | YP_009600218 | Hypothetical protein | 21 | 116 | ORF3015 | WP_012180164 | Phytoene desaturase | 240 | 329 | 29.59 | 9.00E-03 |
| ORF74 | YP_009600220 | DNA polymerase I | 81 | 670 | ORF2493 | WP_012179570 | DNA polymerase I | 352 | 931 | 27.03 | 5.75E-45 |
| ORF83 | YP_009600229 | Hypothetical protein | 79 | 159 | ORF3999 | WP_245533003 | Response regulator | 666 | 744 | 26.37 | 1.60E-02 |
| ORF67 | YP_009600213 | Exodeoxyribonuclease-5 | 1 | 400 | ORF2444 | WP_012179511 | AAA family ATPase | 6 | 477 | 26.3 | 9.55E-20 |

| | | | | | | | | | | | |
|---|---|---|---|---|---|---|---|---|---|---|---|
| ORF55 | YP_009600201 | ATP-dependent DNA helicase | 7 | 285 | ORF3598 | WP_044027873 | UvrD-helicase domain-containing protein | 53 | 346 | 25.08 | 2.86E-15 |
| ORF49 | YP_009600195 | Thymidylate synthase | 76 | 263 | ORF0683 | WP_012177445 | Thymidylate synthase | 74 | 255 | 24.37 | 6.69E-05 |
| ORF87 | YP_009600233 | ATP-dependent DNA helicase | 17 | 381 | ORF3598 | WP_044027873 | UvrD-helicase domain-containing protein | 46 | 413 | 22.39 | 7.82E-07 |
| ORF50 | YP_009600196 | Superfamily II DNA/RNA helicase | 446 | 546 | ORF1171 | WP_012178005 | DEAD/DEAH box helicase | 257 | 348 | 21.78 | 3.10E-02 |

*predicted by BLASTp, the threshold of e-value is 0.05.

**Table S5.** List of 32 freshwater cyanophages that have been experimental isolated and genome-sequenced.

| Host | Phage | Morphology | Genome size (kb) | Accession number | References |
|---|---|---|---|---|---|
| *Pseudanabaena* | PA-SR01 | Tailless | 137.01 | MT234670 | Zhang, D. et al., 2020 |
| | Pam1 | *Podoviridae* | 36.04 | ON014753 | Du, K. et al., 2022 |
| | Pam2 | *Siphoviridae* | 142.86 | ON014754 | Du, K. et al., 2022 |
| | Pam3 | *Myoviridae* | 54.54 | ON014755 | Du, K. et al., 2022 |
| | Pam4 | *Podoviridae* | 48.35 | ON014756 | Du, K. et al., 2022 |
| | Pam5 | *Siphoviridae* | 39.51 | ON014757 | Du, K. et al., 2022 |
| *Microcystis* | Ma-LMM01 | *Myoviridae* | 162.10 | NC_008562 | Yoshida, T. et al., 2008 |
| | MaMV-DC | *Myoviridae* | 169.20 | NC_029002 | Ou, T. et al., 2013 |
| | Mic1 | *Siphoviridae* | 92.63 | MN013189 | Yang, F. et al., 2020 |
| | Me-ZS1 | *Siphoviridae* | 49.67 | MK069556 | Lin, W. et al., 2020 |
| | PhiMa05 | *Myoviridae* | 27.39 | MW495066 | Naknaen, A. et al., 2021 |
| | MinS1 | *Siphoviridae* | 49.97 | MZ923504 | Zhang, S. et al., 2022 |
| | Mea-Yong924-1 | Unassigned | 40.33 | MZ447863 | Qian, M. et al., 2022 |
| *Synechococcus* | S-2L | *Siphoviridae* | 45.50 | MW334946 | Marliere, P. et al., 2006 |
| | S-CRM01 | *Myoviridae* | 178.56 | NC_015569 | Dreher, T. W. et al., 2011 |
| | S-EIV1 | *Podoviridae* | 79.18 | KJ410740 | Chenard, C. et al., 2015 |
| | S-LBS1 | *Siphoviridae* | 34.64 | MG271909 | Zhong, K. X. et al., 2018 |
| | B3 | *Myoviridae* | 244.93 | MN695334 | Levesque, A. V. et al., 2020 |
| | B23 | *Myoviridae* | 243.63 | MN695335 | Levesque, A. V. et al., 2020 |
| | S-SRM01 | *Myoviridae* | 240.84 | MW015081 | Zhang, D. et al., 2021 |
| | S-SRP01 | *Podoviridae* | 45.02 | MW015080 | Zhang, D. et al., 2021 |
| | S-SRP02 | *Podoviridae* | 42.14 | MW822601 | Zhang, D. et al., 2022 |
| *Phormidium* | Pf-WMP4 | *Podoviridae* | 40.49 | DQ875742 | Liu, X. et al., 2007 |
| | Pf-WMP3 | *Podoviridae* | 43.20 | NC_009551 | Liu, X. et al., 2008 |
| | PP* | *Podoviridae* | 42.48 | NC_022751 | Zhou, Y. et al., 2013 |
| *Anabaena* | A-4L | *Podoviridae* | 41.75 | NC_024358 | Ou, T. et al., 2015 |
| | A1 | *Myoviridae* | 68.30 | KU234533 | Chenard, C. et al., 2016 |
| | N1 | *Myoviridae* | 64.96 | KU234532 | Chenard, C. et al., 2016 |
| *Raphidiopsis* | CrV-01T | *Siphoviridae* | 104.36 | MH636380 | Martin, R. M. et al., 2018 |
| | Cr-LKS3 | *Siphoviridae* | 46.25 | OM373202 | Laloum, E. et al., 2022 |
| *Planktothrix* | PaV-LD | Tailless | 95.30 | NC_016564 | Gao, E. B. et al., 2012 |
| *Arthronema* | TR020 | *Podoviridae* | 44.81 | MT457475 | Petrzik, K. et al., 2020 |

*Cyanophage PP could also infect cyanobacteria *Plectonema*.
